# Bioleaching of E-Waste: Influence of Printed Circuit Boards on the Activity of Acidophilic Iron-Oxidizing Bacteria

**DOI:** 10.3389/fmicb.2021.669738

**Published:** 2021-08-18

**Authors:** Juan Anaya-Garzon, Agathe Hubau, Catherine Joulian, Anne-Gwénaëlle Guezennec

**Affiliations:** ^1^Bureau de Recherches Géologiques et Minières, Orléans, France; ^2^Chimie ParisTech, PSL Research University, CNRS, Institut de Recherche de Chimie Paris, Paris, France

**Keywords:** iron-oxidizing bacteria, *Leptospirillum ferriphilum*, printed circuit boards, bioleaching, pre-oxidation phase, subculturing

## Abstract

Bioleaching is a promising strategy to recover valuable metals from spent printed circuit boards (PCBs). The performance of the process is catalyzed by microorganisms, which the toxic effect of PCBs can inhibit. This study aimed to investigate the capacity of an acidophilic iron-oxidizing culture, mainly composed of *Leptospirillum ferriphilum*, to oxidize iron in PCB-enriched environments. The culture pre-adapted to 1% (w/v) PCB content successfully thrived in leachates with the equivalent of 6% of PCBs, containing 8.5 g L^–1^ Cu, 8 g L^–1^ Fe, 1 g L^–1^ Zn, 92 mg L^–1^ Ni, 12.6 mg L^–1^ Pb, and 4.4 mg L^–1^ Co, among other metals. However, the inhibiting effect of PCBs limited the microbial activity by delaying the onset of the exponential iron oxidation. Successive subcultures boosted the activity of the culture by reducing this delay by up to 2.6 times under batch conditions. Subcultures also favored the rapid establishment of high microbial activity in continuous mode.

## Introduction

Printed circuit boards (PCBs) are the main processing unit for the electronic devices that have become essential to our daily lives in recent decades. Their disposal at the end of their useful life is a major challenge for the circular economy and sustainable development. Improper handling of waste PCBs is widespread, with incineration and landfilling as the easiest but polluting solutions. Waste PCBs contain heavy metals (Pb, Hg, As, Cd, Cr), polychlorinated biphenyls, polybrominated biphenyls, and epoxy resins, which are highly toxic for the environment (Kaya, 2019). Likewise, spent PCBs are a valuable source of various strategic metals, including Au, Ag, Pd, Ti, Ga, Ta, Co, Cu, Fe, Al, Zn, Sn, Pb, and Ni (Hubau et al., 2019; Kaya, 2019).

The dissolution of metals assisted by microorganisms, also known as bioleaching, is a biotechnological process that is now established worldwide. This technology is already applied at an industrial scale for the extraction of Au and Cu, and marginally for Ni, Co, and U, from primary ores (Rawlings and Johnson, 2007; Wassink and Asselin, 2019). It is now attracting considerable attention for the extraction of metals from secondary sources such as mining waste, contaminated river sludge (Zhang et al., 2018), fly ash (Xu et al., 2014), batteries (Liu et al., 2020), and spent PCBs (Bryan et al., 2015, 2020; Guezennec et al., 2015; Işıldar et al., 2016; Wu et al., 2018; Hubau et al., 2020). Some of the advantages of biotechnologies compared to conventional pyrometallurgical and hydrometallurgical recycling processes are the safety conditions for operators, operations at atmospheric pressure and room temperature, lower chemical and energy consumption, lower CO_2_ emissions, and low initial investments (Baniasadi et al., 2019). Over the last decade, bioleaching of PCBs has been investigated at the research level in shake flasks (Adhapure et al., 2013; Arshadi and Mousavi, 2015; Bryan et al., 2015; Guezennec et al., 2015) and stirred tank reactors (STR) with capacities above 1 L (Ilyas and Lee, 2014; Guezennec et al., 2015; Mäkinen et al., 2015; Xia et al., 2017; Hubau et al., 2020).

Microorganisms employed in the bioleaching of PCBs are diverse, with both autotrophs (Xiang et al., 2010) and heterotrophs (Chi et al., 2011) and mesophilic and thermophilic (Ilyas et al., 2007) species. Chemolithotrophs acidophilic iron-oxidizing bacteria (FeOB) stand out among the group of microorganisms most frequently studied for dissolving metals (Xiang et al., 2010; Işıldar et al., 2019; Hubau et al., 2020). Ferric iron and sulfuric acid act as oxidizing agents to mobilize the metals from the solid matrix (Eqs 1, 2). FeOB uses the resulting Fe(II) as a source of energy to regenerate the ferric iron in the presence of dissolved oxygen (Eq. 3), which accelerates the bioleaching process.

(1) $$n\,F\,e^{3+}+M\,e\to n\,F\,e^{2+}\,M\,e^{n+}$$

(2) $$n\,M\,e+2\,n\,H^{+}+0.5\,n\,O_{2}\to n\,M\,e^{n+}+n\,H_{2}\,O$$

(3) $$2\,F\,e^{2+}+0.5\,O_{2}+2\,H^{+}\overset{B\,a\,c\,t\,e\,r\,i\,a}{⟶}2\,F\,e^{3+}+H_{2}\,O$$

*Leptospirillum* can potentially thrive in bioleaching environments as they have a greater capacity for iron oxidation activity than other FeOBs (Rawlings et al., 1999; Kinnunen and Puhakka, 2004; Rawlings, 2005; Bryan et al., 2011; Guezennec et al., 2015). These acidophilic and moderately thermophilic species are highly resistant to environments containing high concentrations of Fe(III) and can survive high concentrations of other metals (Dopson and Holmes, 2014). Dopson and Holmes (2014) describe possible passive and active mechanisms these microorganisms might use to grow in the presence of a high metal content. *Leptospirillum ferriphilum* is a dominant FeOB in industrial continuous-flow biooxidation tanks for mineral extraction (Rawlings et al., 1999; Coram and Rawlings, 2002). These Gram-negative bacteria have a large number of genes related to heavy metal detoxification, oxidative stress defense, DNA repair, pH tolerance to acidic environments, and signal transduction to adapt to the external environment (Mi et al., 2011). However, one of the major challenges in PCB bioleaching remains the inhibiting effects of the waste material on the metabolism of these microorganisms (Guezennec et al., 2015; Wu et al., 2018; Arya and Kumar, 2020). PCBs can slow down/delay microbial growth and reduce microbial capacity to oxidize iron (Edward et al., 2018). Consequently, the regeneration of Fe(III) decreases along with leaching rates. It is not clear whether the toxic effect results from the metal content, the organic content of PCBs, or both (Bryan et al., 2015; Baniasadi et al., 2019). Yang et al. (2014) suggest that Pb, Ni, and plastics have inhibiting effects on bacterial growth, but it is not evident whether there is a main limiting factor. Besides, a synergy between different metal ions can increase the level of toxicity compared to the individual effect of each metal, as described by Nurmi et al. (2009). Finally, damage to bacterial cells can also result from attrition of the bacterial membrane by solid particles (Haghshenas et al., 2009).

Different strategies have been proposed to tackle the toxicity effect of PCB bioleaching processes. The first strategy consists of separating biogenic Fe(III) production from the PCB leaching (Brandl et al., 2001; Zhu et al., 2011; Guezennec et al., 2015; Wang et al., 2018; Hubau et al., 2020), which alloys microorganisms to thrive in a system without the toxic effect of PCBs. This strategy can be employed in continuous flow bioreactors to achieve high bioleaching efficiencies over short periods. Hubau et al. (2020) showed high PCB bioleaching efficiency in steady-state conditions within 48 h and 1% (w/v) PCBs. However, a long transitional regime of several months was required to achieve high and stable microbial activity.

The second strategy seeks to adapt microorganisms to the toxic waste material to improve the iron-oxidizing activity and trigger leaching efficiency (Rawlings, 2005; Haghshenas et al., 2009; Yang et al., 2009, 2014; Hubau et al., 2020). A typical method involves subculturing in a specific environment. Serial subculturing can, for instance, enhance tolerance to a high metal content and improve microbial iron oxidation activity (Bryan et al., 2011; Liu et al., 2019). The period before the microbial oxidation of iron, referred here as the pre-oxidation phase, can be used as an indicator of microbial iron oxidation activity (Das et al., 1998; Haghshenas et al., 2009). This pre-oxidation phase reflects the time required for bacteria to start actively catalyzing iron oxidation. In this sense, it is possible to estimate the evolution of bacterial tolerance to PCBs by means of serial subcultures following the pre-oxidation phase.

The main objective of this study was to investigate the capacity of FeOB cultures dominated by *L. ferriphilum* to grow and improve the iron oxidation kinetics in PCB-enriched environments. The inhibiting effects of PCBs on microorganisms were covered (i) by studying the inhibiting effect of chemically dissolved PCB metals on the microbial growth and activity of a FeOB culture in shake flasks and (ii) by investigating the microbial growth and oxidizing activity of two cultures referred to as “adapted” and “non-adapted” in a batch STR at different PCB solid loads. Strategies to tackle this inhibition were then investigated. They rely on (i) successive subcultures in batch mode and (ii) different operational modes to reach the steady state in continuous mode, as fast as possible and with high microbial activity.

## Materials and Methods

### Bacterial Culture

This study used two FeOB cultures, referred to here as “adapted” and “non-adapted” cultures. Both come from a two-step bioprocess for PCB bioleaching, which is fully described in previous studies (Hubau et al., 2018, 2020). The non-adapted culture, which came from a 150-mL bio-oxidation aerated column, run in continuous mode and inoculated with an acidophilic FeOB culture, showed highly efficient oxidation of Fe(II) at 1 g L^–1^ h^–1^, but was never in contact with PCBs. This culture was mainly made up of *L. ferriphilum* and *Sulfobacillus benefaciens*, with *L. ferriphilum* being the predominant species (Hubau et al., 2018). This ferric iron-rich lixiviant was used to feed a PCB-bioleaching STR, operated in continuous mode with 1% (w/v) PCBs for over 4 months at a hydraulic retention time (HRT) of 48 h. The adapted culture came from a leachate of the bioleaching reactor. High metal dissolution yields and rates were obtained under these conditions (Hubau et al., 2020). *L. ferriphilum* was the predominant bacterium in both cultures (Hubau et al., 2018, 2020).

The 0Cm medium used in this study is derived from 0Km with a reduced (NH_4_)_2_SO_4_ concentration (Hubau et al., 2018) and has previously enabled a robust performance of Fe(II) bio-oxidation in the aerated column and a high bioleaching yield in the STR (Hubau et al., 2020). This medium is composed of 0.4 g L^–1^ (NH_4_)_2_SO_4_ (extra pure, Merck, Darmstadt, Germany), 0.81 g L^–1^ H_3_PO_4_ (analytical grade, Merck), 0.48 g L^–1^ KOH (pure pellets, Merck), and 0.52 g L^–1^ MgSO_4_.7H_2_O (analytical grade, Merck). The medium was supplemented with 3 g L^–1^ of Fe(II) as FeSO_4_.7H_2_O (99+%, Acros Organics, Fair Lawn, NJ, United States) and was thus denoted 3Cm. The pH was adjusted to 1.2 with H_2_SO_4_ (>95%, analytical grade, Fisher Chemical, Waltham, MA, United States).

### PCB Composition and Preparation

Samples of low-grade PCBs were collected from small appliances at the *Envie 2E Midi-Pyrenees* WEEE sorting center (France). Preparation of the PCBs and determination of the metal composition are fully described in Hubau et al. (2019). Briefly, the PCBs were shredded to reduce the particle size below 750 μm (750 μm corresponding to the mesh size of the shear shredder Bohmier Maschinen GmbH) and a laboratory knife mill (Retsch, 2000). Table 1 shows the metal composition of the samples (Hubau et al., 2019).

**TABLE 1:** Element composition in % weight of low-grade PCB samples.

**Element**	**Concentration (% weight)**	**Element**	**Concentration (% weight × 10^–5^)**
Cu	14.58	Cr	842
Fe	12.23	Co	358
Al	6.04	Ag	209
Zn	1.67	Mo	152
Sn	1.67	In	100
Pb	1.17	Au	44
Mn	0.61	Pd	25
Ni	0.34	V	22
Sb	0.20	W	15
Mg	0.14	Ga	12
		Ta	7.5
		Ge	0.75
		Pt	0.59

A solution of dissolved PCB metal ions was prepared by abiotic chemical leaching of 10% (w/v) PCBs in a stirred tank bioreactor with a working volume of 2.2 l. The setup of the STR was previously presented by Hubau et al. (2020). The chemical leaching was performed in the 0Cm culture medium with 10 g L^–1^ of Fe(III) added as Fe_2_(SO_4_)_3_.xH_2_O as a leaching agent in sulfuric acid media and adjusted to pH 1.2 with H_2_SO_4_ (>95%, analytical grade, Fisher Chemical). An airflow rate of 90 L h^–1^ injected beneath the turbine at the bottom of the reactor via a stainless-steel pipe provided with surplus oxygen to facilitate the leaching of metals. A dual (axial/radial) impeller system in stainless steel ensured the mixing of gas, liquid, and solids with a stirring speed of 650 rpm. The temperature of the reactor was maintained at 35°C with a thermostatic bath (Lauda), and the pH was daily adjusted to 1.2 with H_2_SO_4_ until the end of the experiment. Once the metal concentrations were stable (see section “Analytical Techniques”), the solution was filtered.

### Microbial Performance at Different Concentrations of PCB Leachate

The microbial growth and activity of the adapted culture were evaluated in 100-mL (working volume) shake flasks. The experiments contained 20% (v/v), 40% (v/v), 60% (v/v), or 80% (v/v) of the chemical PCB leachate (Table 2), which represented the equivalent of 2, 4, 6, and 8% of PCBs, respectively. Each flask had 10% (v/v) inoculum, the 0Cm culture medium (supplemented with a 10-fold solution of concentrated nutrients), and 8 g L^–1^ Fe(II) (adjusted with a 10-g L^–1^ Fe(II) solution). All solutions were adjusted to pH 1.2 at the beginning of the experiments. The experiments were run in triplicate, with two control duplicates: 10% (v/v) inoculum without PCB leachate, and uninoculated 80% (v/v) PCB leachate. The cultures were incubated in a rotary shaker at 107 rpm and 35°C. The initial microbial concentration was 10^7^ cells mL^–1^. Experiments were run for 25 days, with pH, redox potential, and bacterial concentration monitored daily (See section “Analytical Techniques”). Water losses by evaporation were compensated with sterile distilled water.

**TABLE 2:** Metal concentration of the chemical PCB leachate used to test the microbial performance in shake flasks.

**Element**	**Concentration (g L^–1^)**	**Element**	**Concentration (g L^–1^)**	**Element**	**Concentration (g L^–1^)**
Cu	15.2	Mg	0.069	Pb	0.006
Fe	12.8	Mn	0.046	Co	0.006
Al	3.89	Sn	0.022	V	0.001
Zn	2.21	Cr	0.008	Sb	0.001
Ni	0.22				

*Leaching conditions: 10% (w/v) PCBs, 10 g L^–1^ Fe, Ag, Au, Mo, In, Pd, W, Ga, Ta, Ge, and Pt were below the detection limit (1 μg L^–1^).*

### Microbial Performance During Bioleaching at Different Concentrations of Solid PCBs

The microbial growth and iron oxidation activity of the adapted and non-adapted cultures were evaluated in a 2.2-L STR with shredded solid PCBs at 1% (w/v) or 2% (w/v) in batch mode. First, bacteria were inoculated at 10% (v/v) in 3Cm medium (containing 3 g L^–1^ Fe(II) and acidified to pH 1.2) without PCBs. This step ended when the redox potential reached values above 800 mV [adjusted to be relative to a standard hydrogen electrode (SHE)], i.e., when Fe(II) was oxidized (See section “Analytical Techniques”). PCBs were subsequently added at either 1% (w/v) PCBs or 2% (w/v) PCBs. pH was daily adjusted to 1.2 ± 0.1 with concentrated H_2_SO_4_. pH, redox potential, planktonic cell concentration, and metal concentration were monitored over time.

The same reactor used for the chemical leaching of PCBs was employed for these experiments. An air flow of 60 L h^–1^ enriched with 1% CO_2_ was maintained, to ensure the carbon supply for microbial growth and iron oxidation activity (Guezennec et al., 2018). STR stirring speed was set at 650 rpm, and the reactor was maintained at 35°C.

### Subculturing Protocol in STR

The subcultures were studied in a two-step process, similar to the one described in the previous section, in the 2.2-L STR configuration in batch mode.

**FIGURE 1:**
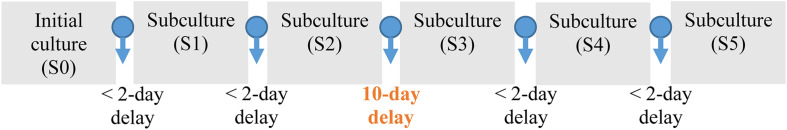
Scheme of the successive subculturing procedure applied on 2.2 L-STR in batch conditions. Each culture contained 3Cm medium and 2% (w/v) PCBs.

In the first experiment (S0), the batch reactor was inoculated with 10% (v/v) of the adapted culture in a 3Cm medium as first step. This step ended when the redox reached values above 800 mV vs. SHE. In the second step, 2% (w/v) PCBs were added to the reactor and the pH was daily adjusted to 1.2 with concentrated H_2_SO_4_. The test was ended when the redox again reached values above 800 mV vs. SHE. The subculture batch reactors were inoculated with 10% (v/v) from the previous test into a 3Cm medium, and the protocol as in S0 was repeated. A diagram of the subculture procedure is presented in Figure 1. Subcultures named S1 and S2 were successively inoculated with an interval of 1–2 days between subcultures to assure that stable conditions were reached. After the second subculture (S2), the culture was stored at room temperature in a hermetic and sterile plastic vessel for 10 days in a basement without light. Subsequently, subcultures S3, S4, and S5 were carried out as in S0. Bacterial cells at the beginning and at the end of each experiment were counted in an optimal microscope as detailed in the section “Analytical Techniques.” This quantitative method was as indicator of microbial growth. No changes were observed in the bacterial concentration after the storage period.

### Start-Up Scenarios in Continuous Mode

For these experiments, we employed a two-stage bioleaching process in continuous flow. The first stage is the aerated column with non-adapted bacteria feeding the 2.2-L STR containing the PCBs as the second stage. The aerated column contained a solid support on polypropylene for bacterial growth, while no support was included in the second stage (PCB bioleaching STR). The setup is described in detail in a previous study (Hubau et al., 2020). Three scenarios were proposed to evaluate the microbial iron oxidation activity during the start-up of the continuous process and to determine a strategy to reach the steady state as fast as possible.

In the first scenario (I), the 2.2-L STR reactor was progressively filled with the ferric iron lixiviant (generated in the aerated column), containing non-adapted bacteria and 3 g L^–1^ of Fe(III) at redox potential above 800 mV vs. SHE. The output flow from the aerated column continuously fed the STR at 47 mL h^–1^ with a pump to maintain an HRT of 48 h. PCBs were added once per hour at 0.45 g h^–1^ to maintain a PCB load of 1% (w/v). Agitation started after 24 h. After 48 h, when the working volume reached 2.2 L, pumping of the effluent began.

In the second scenario (II), a test in batch mode was performed with adapted bacteria as described in the subculturing protocol. The batch had 10% (v/v) inoculum in a 3Cm medium, and the equivalent of 1% (w/v) PCBs was loaded once the redox reached over 800 mV vs. SHE. The continuous-flow bioleaching mode started at the end of this batch with the total pulp in the reactor. The aerated column effluent and PCBs fed the reactor continuously at 48 h of HRT and 1% (w/v) PCBs as in the first scenario.

In the third scenario (III), an initial bioleaching batch process was performed with 10% (v/v) of the adapted bacteria and 2% (w/v) PCBs, followed by two successive batch subcultures. Each subculture was inoculated with 50% (v/v) from the previous batch. The other 50% volume contained 3Cm. One percent (w/v) of PCBs was added after the redox rose above 800 mV vs. SHE in each subculture. After the second subculture, the continuous-flow mode started. As in scenarios I and II, the reactor worked with an HRT of 48 h and 1% (w/v) PCBs and was fed with non-adapted bacteria from the aerated column.

All the bioleaching reactors in batch and continuous-flow modes operated at 35°C, with an airflow enriched with 1% CO_2_ of 60 L h^–1^, and agitation at 650 rpm. The pH was maintained lower than 1.6 to avoid Fe(III) precipitation. The start-up phase was considered finished when the redox potential maintained values above 800 mV vs. SHE in continuous mode for at least two HRT.

### Analytical Techniques

The pH values and redox potentials were monitored daily for all experiments with pH (IDS SenTix^®^ 940) and redox (BlueLine 32-3 RX IDS) electrodes, respectively, both connected to a multiparameter portable meter (Multi 3630 IDS, WTW). Metals were analyzed with a Varian SpectrAA 300 flame atomic absorption spectroscope (FAAS). The oxidation of iron was deduced from the correlation of Yue et al. (2016) expressed in Eq. 4. This involves the redox potential (*Eh* in mV vs. SHE), the temperature (*T* in K), the number of electrons exchanged (*n*), the gas constant (*R* = 8.3 J K^–1^ mol^–1^) and the Faraday constant (*F* = 96485 C mol^–1^), and the concentration of Fe (*[Fe]* in mg L^–1^).

(4) $$E\,h\,10-3\,x\,{\left[T\right]}^{2}+0,91\,T+2,303\,x\,10^{3}\,\frac{R\,T}{n\,F}\,l\,o\,g\,\frac{\left[F\,e\,\left(I\,I\,I\right)\right]}{\left[F\,e\,\left(I\,I\right)\right]}+492$$

To estimate the microbial growth, 20-μL samples of the leachate were placed in a Thoma cell counting chamber and observed through an optical microscope at ×400 magnification. This technique shows the growth tendency of planktonic bacteria. The diversity of the bacterial (or prokaryotic) consortium was characterized using the capillary electrophoresis single-strand conformational polymorphism (CE-SSCP) molecular fingerprinting technique on the 16S rRNA gene encoding, using the protocol described by Hubau et al. (2018).

## Results

### Microbial Performance at Different Leachate Concentrations

These experiments performed in shake flasks aimed to characterize the bacterial performance of the adapted FeOB culture in PCB environments. The PCB environment was reproduced from a concentrated PCB leachate, whose metal concentration is detailed in Table 2. The data show that the PCB leachate contained mainly Cu, Fe, Al, Zn, and Ni. A wide range of other metals was present in concentrations below 100 mg L^–1^.

**FIGURE 2:**
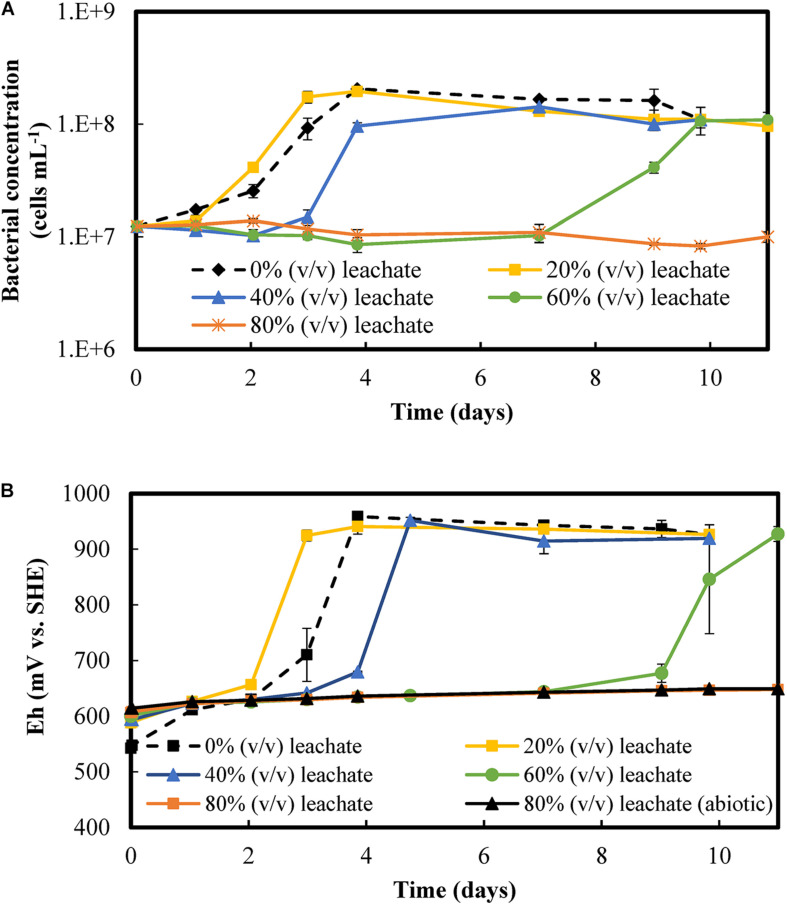
Microbial activity at various concentrations of the chemical leachate produced with 10% (w/v) PCBs: **(A)** Bacterial concentration and **(B)** redox potential. The experiments were performed in shake flasks and started with 8 g L^– 1^ Fe(II) and 10^7^ cells mL^− 1^. The error bars represent the range of all values obtained under fixed operating conditions. Additional information on experiments with 80% (v/v) leachate the abiotic control is available up to day 25, although no significant changes in either redox behavior or bacterial concentration were observed.

The effect of increased concentrations of metal-rich PCB leachates on bacterial growth and Fe(II)-oxidizing activity is presented in Figure 2. Figure 2A shows that the lag phase lasted longer at increased concentrations of PCBs, with 1 day at 20% (v/v) of PCB leachate, 2 days at 40% (v/v) of PCB leachate, and 7 days at 60% (v/v) of PCB leachate. Starting from 10^7^ cells mL^–1^, the bacterial concentration increased exponentially and reached a stationary phase at 10^8^ cells mL^–1^ in conditions containing up to 60% (v/v) of PCB leachate. A similar behavior was observed in the biotic control without dissolved PCBs, which had a lag phase of 1 day. The control assay with 80% (v/v) of leachate (corresponding to 8% of PCBs) did not present a microbial growth over the 25 days of experiments, as verified through bacterial counting.

Figure 2B shows that the redox potential increased simultaneously with the exponential microbial growth when the solutions contained 20% (v/v) to 60% (v/v) of PCB leachate. The duration of the lag phase and the pre-oxidation phase were equal under these conditions, indicating that substrate oxidation and microbial growth were correlated. At the end of the pre-oxidation phase, the redox increased exponentially from less than 650 mV vs. SHE to more than 900 mV vs. SHE in at most 2 days. This redox behavior can be attributed to the iron oxidation catalyzed by microorganisms and continued to a plateau, which corresponds to a complete oxidation of iron according to Eq. 4. In the biotic control without leachate, the microbial iron oxidation and the stationary microbial growth phase took 1 day longer than in the assay with 20% (v/v) of PCB. At 80% (v/v) of PCB leachate, the redox gradually increased over time, representing 37% of iron oxidized in 25 days regardless of the presence of bacteria. Therefore, no bacterial growth or iron oxidation related to the microbial iron oxidation activity were found during this period at 80% (v/v) of PCB leachate.

Despite the delay of 7 days in microbial growth and iron oxidation activity, the culture thrived in a medium with a concentration of metals six times higher than in the medium to which they were initially adapted. Based on the initial leachate concentration (Table 2), the solution at 60% (v/v) of PCB leachate contained 9 g L^–1^ Cu, 2 g L^–1^ Al, 1 g L^–1^ Zn, 0.1 g L^–1^ Ni, 10 mg L^–1^ Pb, and 4 mg L^–1^ Co, among other elements. According to the molecular fingerprinting test, *L. ferriphilum* is the predominant species whatever the leachate concentration, as in the test with no leachate (Supplementary Figure 1). This confirms the capacity of *L. ferriphilum* to grow and oxidize iron at high concentrations of mixed metals and other elements.

### Effects of Solid PCBs on Microbial Performance During Bioleaching

The purpose of these experiments was to investigate the inhibiting effect of solid PCBs on the microbial growth and pre-oxidation phase. In the experiments, metal leaching (Eqs 1, 2) and the microbial oxidation of Fe(II) to regenerate Fe(III) as a leaching agent (Eq. 3) occurred in the same reactor.

Table 3 presents the final planktonic cell concentration and the duration of the pre-oxidation phase of cultures in the presence of 1% (w/v) PCBs and 2% (w/v) PCBs. The tendency of planktonic bacteria to grow in bioreactors with 2% (w/v) PCBs seems higher compared to reactors with 1% (w/v) PCBs. There could be an increased supply of iron for microbial growth thanks to the iron dissolution from 2% (w/v) PCBs. However, this result must be considered carefully since the addition of PCBs may interfere with the counting of bacterial cells. Besides, a considerable proportion of biomass can attach to the solids. The effects of PCB load on microbial growth were therefore difficult to measure in these conditions.

**TABLE 3:** Final planktonic cell concentration and duration of the pre-oxidation phase in 2.2-l STR PCB bioleaching reactors in batch mode.

	**Non-adapted culture**		**Adapted culture**	
	**1% (w/v) PCB**	**2% (w/v) PCB**	**1% (w/v) PCB**	**2% (w/v) PCB**
Final planktonic cell concentration (cells mL^–1^)	10^7^	6 × 10^7^	3 × 10^7^	10^8^
Pre-oxidation phase during PCB bioleaching (days)	>16^*a*^	29	11	18

*^*a*^No exponential rise of the redox observed in 16 days.*

*The planktonic cell concentration before the addition of PCBs was 10^7^ cells mL^–1^.*

On the other hand, the increase in the PCB load prolonged the pre-oxidation phase, as in the previous experiments with the PCB leachates (Figure 2). The onset of microbial iron oxidation took 11 days at 1% (w/v) PCBs and 18 days at 2% (w/v) PCBs with the adapted culture (Supplementary Figure 2). This pre-oxidation phase took consistently longer compared to the shake flask tests containing PCB leachates (Figure 2). Despite the different configuration, the presence of solids in the bioleaching experiments may have affected the microbial iron oxidation due to the presence of toxic solid elements such as plastics, resins, and heavy metals.

The evolution of metal concentration and dissolution yield over time is presented in Supplementary Figure 3. The bioleaching system took 7 days to reach a maximum dissolution yield of 100% Cu, 70% Ni, and 100% Zn, regardless of the solid load and the microbial culture. Leachates of 1% (w/v) PCBs reached a maximum of 2.6 g L^–1^ Cu, 30 mg L^–1^ Ni, and 303 mg L^–1^ Zn, whereas leachates of 2% (w/v) PCBs contained up to 4.2 g L^–1^ Cu, 52 mg L^–1^ Ni, and 543 mg L^–1^ Zn. The leachates started with 3 g L^–1^ Fe coming from the 3Cm medium and reached up to 3.3 g L^–1^ Fe at 1% (w/v) PCBs and 3.6 g L^–1^ Fe at 2% (w/v) PCBs. The dissolution yield of Fe appears low (below 20%), but it did not consider Fe precipitation (Chen et al., 2015; Hubau et al., 2020).

### Subculturing of Bacteria From PCBs Bioleaching in Batch Conditions

The subculturing experiments in the 2.2-L STR were carried out with the adapted culture to improve the microbial capacity to oxidize iron in PCB environments by shortening the pre-oxidation phase. Each subculture initially contained 10^7^ cells mL^–1^ and led to a final concentration of 10^8^ cells mL^–1^ (for S0, S1, S2) and 2 × 10^8^ cells mL^–1^ (S3, S4, and S5). The bacterial concentration in the liquid phase increased exponentially in the first step (absence of PCBs) and continued to grow slowly afterward with no well-defined lag or exponential phases (Supplementary Figure 4).

**FIGURE 3:**
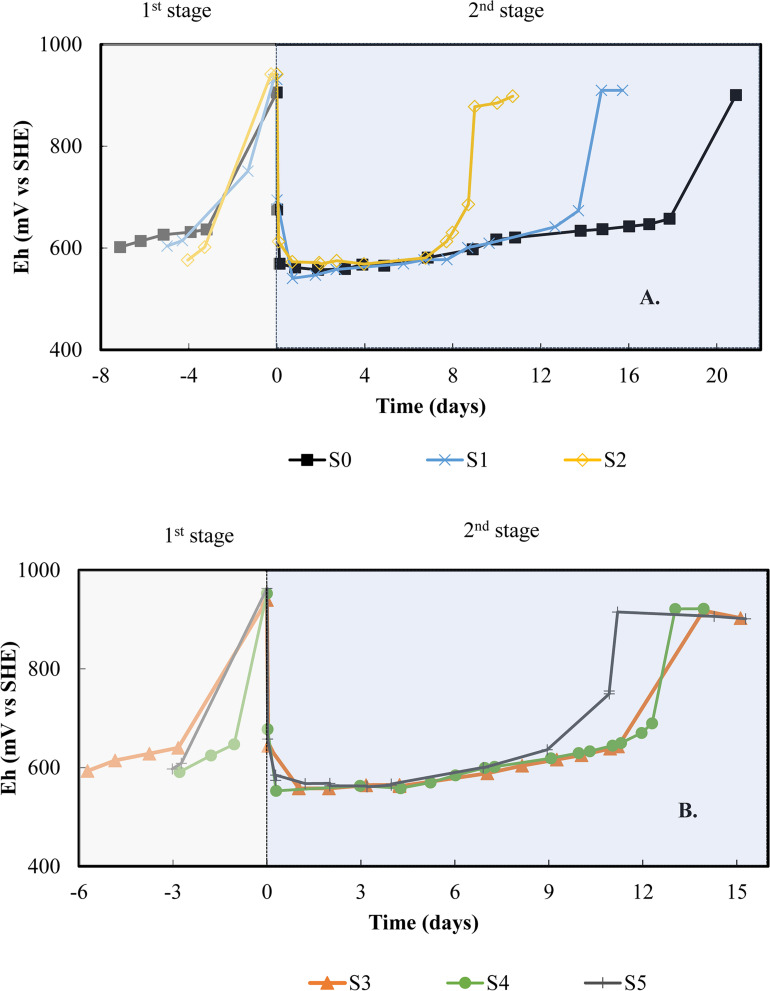
Follow up of redox potential in bioleaching batch subcultures in 2.2 L-STRs: **(A)** subcultures SO, SI, and S2, **(B)** subcultures S3, S4, and S5. The first stage involves the microbial oxidation of the 3 Cm medium while the second stage starts with the addition of 2% (w/v) PCBs.

Figure 3 shows the redox behavior of each subculture during the first stage with 3Cm medium and the second stage with the PCBs. The reference point or day 0 is considered to be the moment when 2% (w/v) PCBs were added to the system. Before that reference point, the redox increased from 600 mV vs. SHE to over 900 mV vs. SHE within 3–7 days depending on the subculture. At this point, 3 g L^–1^ of Fe were in ferric form. Drops in redox potential of around 400 mV were observed in every batch within the first hour after adding PCBs, indicating that Fe(III) was being reduced. Subsequently, a gradual increase in redox was followed by an exponential rise and a plateau phase as in the presence of PCB leachates without solids (Figure 2). All the subcultures showed an exponential rise of 250–300 mV in approximately 2 days, reaching values close to 900 mV vs. SHE. At the plateau phase, iron was considered to be completely oxidized.

After the addition of PCBs, the subcultures improved the bio-oxidation of Fe(II) by shortening the pre-oxidation phase over time. This decreased from 18 days for the first batch (S0), to 13 for the first subculture (S1), then 7 days for the second subculture (S2), as shown in Figure 3A. The pre-oxidation phase for S2 was therefore 2.6 times faster than for S0. Subsequently, the effect of storing the culture between two subcultures was tested with a 10-day pause between S2 and S3 (Figure 1). The pre-oxidation phase of S3 lasted longer (11 days), but further subcultures S4 and S5 were able to reduce this pre-oxidation phase to 10 and 7 days, respectively (Figure 3B). A similar pattern was observed in the first stage, but no pre-oxidation phase values are given since the redox was not monitored every day.

The evolution of the metal content in the leachate (Supplementary Figure 5) showed a maximum dissolution yield after 7 days as in the previous experiments, regardless of the subculture. At the end of the experiments, the leachate contained, on average, 4.5 g L^–1^ Cu, 50 mg L^–1^ Ni, and 550 mg L^–1^ Zn, among other elements.

### Start-Up Scenarios in Continuous Mode

Continuous mode enables complete dissolution of PCB metals in a short time (less than 48 h) once the bacteria adapted to the bioleaching environment (Hubau et al., 2020). However, microbial adaptation to PCBs is obtained after a long transitional regime of several months. In these experiments, the capacity of the culture to shorten the nonsteady period of a PCB bioleaching reactor in continuous mode was investigated. Three start-up scenarios were explored to accelerate the transitional regime before the culture achieved high iron oxidation activity.

**FIGURE 4:**
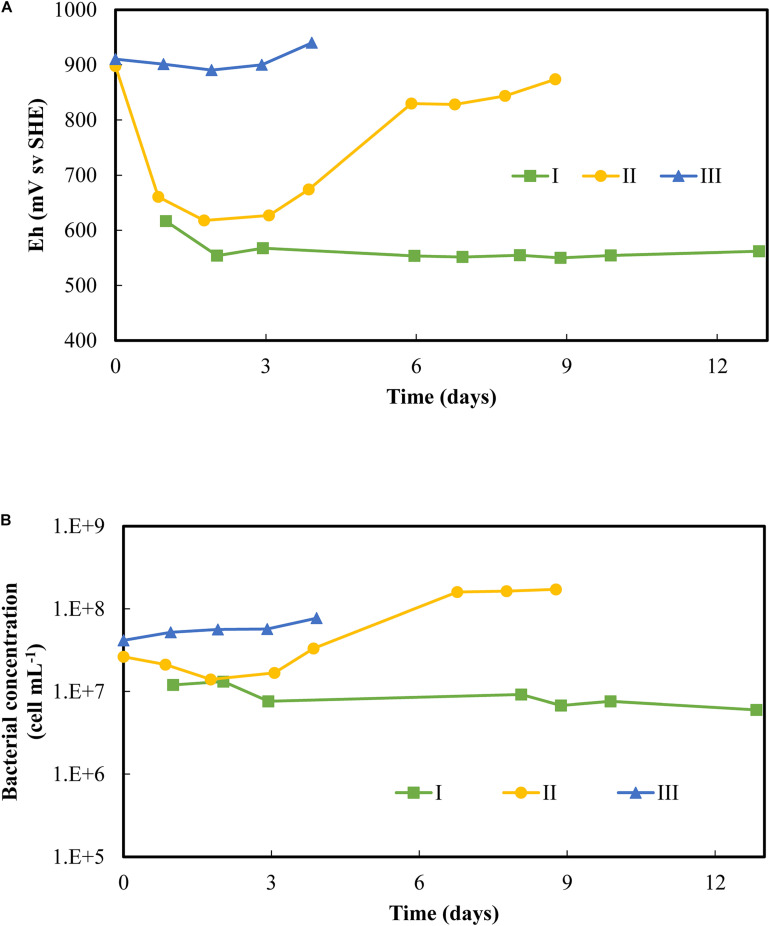
Follow-up of **(A)** redox potential and **(B)** bacterial concentration overtime during the startup of continuous flow PCB bioleaching experiments in 2.2 L-STRs. Three scenarios of the startup were tested: I was only inoculated with the non-adapted culture; II performed a batch test on the adapted culture before starting the continuous flow process; III performed two successive batch subcultures on the adapted culture prior to the continuous flow process. Conditions of continuous flow bioleaching were set at 48 h of HRT and 1% (w/v) PCBs.

Figure 4 shows the evolution of the redox potential and bacterial concentration in the liquid phase during the start-up of the three scenarios. The first scenario (I) starts with non-adapted bacteria from the aerated column output as the only source of inoculum. In scenario II and scenario III, the adapted bacteria were first cultivated in batch mode before being used in PCBs bioleaching CSTR, without and with subcultures, respectively. These batch steps with PCBs lasted 18 days for scenario II and 14 days for scenario III (Supplementary Figure 6). Scenario I maintained a low redox and a constant planktonic bacterial concentration throughout the experiment. Besides, no growth was observed in the liquid phase. In scenario II, the redox was initially high from the previous batch and dropped during the first day as the Fe(III) was rapidly reduced. From the third day, the redox increased simultaneously with microbial growth. After 6 days, the redox reached 830 mV vs. SHE, implying complete Fe(II) oxidation, and the bacterial concentration gradually increased to reach 2 × 0^8^ cells mL^–1^. Scenario III did not show any significant drop in redox over 4 days, equivalent of two HRTs. Iron was therefore maintained as Fe(III) despite the continuous addition of PCBs. At the same time, minor microbial growth was observed (the planktonic bacterial counts increased from 4 × 10^7^ to 8 × 10^7^ cells mL^–1^), but no clear exponential growth was detected.

## Discussion

This study characterized the inhibition of microbial growth in metal-rich leachates and the iron oxidation activity of an adapted FeOB culture dominated by the iron-oxidizing species *L. ferriphilum*. The remarkable ability of *L. ferriphilum* to thrive at very low pH values and high redox potentials is well known (Rawlings et al., 1999; Hedrich et al., 2016). However, its capacity to catalyze iron oxidation can be limited by the inhibiting effect of high PCB concentrations (Arshadi and Mousavi, 2014; Wu et al., 2018). This inhibition can be due to the elevated dissolved metal concentrations, to other nonmetallic elements that may be present in PCBs, and to the solid PCB particles themselves. The FeOB culture adapted to 1% (w/v) PCBs grew and oxidized iron 1 day faster in the presence of 20% (v/v) PCB leachate (equivalent to 2% of PCBs) compared to the biotic control without PCBs. It is difficult to say whether the culture was more efficient when adapted to the leachate in this case or if the values enter in the measurement uncertainty. For media containing up to 60% (v/v) of PCB leachate, the study demonstrates a link between the concentration of the PCB leachate and the delay in bio-oxidation of Fe(II). This study also demonstrated that the addition of leachates [up to 60% (v/v)] had no impact on the final planktonic cell concentration and Fe(II) oxidation yield. In a previous study (Hubau et al., 2020), similar results were obtained with the non-adapted culture, but the bio-oxidation delay was observed at a lower metal content. An improved metal tolerance capacity of the culture adapted to elevated PCB concentrations was likely to be achieved in this study. This is consistent with the study by Edward et al. (2018) who reported uninhibited iron oxidation by a *L. ferriphilum*-dominated culture adapted to 1 g L^–1^ of Cu, achieving full oxidation of 3 g L^–1^ iron in 4 days in the presence of 10 g L^–1^ Cu. The combined inhibitory effects of different metals contained in PCB leachates might explain the impact on microbial activity kinetics in our FeOB culture, as has been observed by Nurmi et al. (2009). Achieving high PCB loads is favorable for the economics of the bioleaching process. However, inhibitions of the microbial cultures have to be taken into account and only non-inhibitory pulp densities can be used.

The batch STR bioleaching experiments confirmed that elevated PCB loads extended the pre-oxidation phase. This delay was noted with both the adapted and non-adapted cultures. There was a pre-oxidation phase of 11 days at 1% (w/v) PCBs, even though the culture was adapted to that environment. The delays observed in the STRs were longer than those in shake flasks. The difference in operating conditions certainly affected the microbial activity, but the prolonged inhibiting effect in the STRs might also be related to (i) the shear stress caused by the solids and (ii) the presence of toxic elements in the solid phase.

A subculturing strategy reduced the inhibiting effect of PCBs in batch STRs. This improvement may be related to the development of microbial resistance mechanisms allowing faster adaptation to the toxic effect of PCBs. It is also consistent with the work of Hedrich et al. (2016), in which successive subcultures improved the bioleaching rate of metals in batch bioleaching reactors. The subculturing strategy in this report achieved a maximum metal dissolution yield in 7 days despite the enhanced iron oxidation activity within subcultures. In all subcultures, the leaching agent was rapidly consumed during the first hour. Therefore, leaching mostly occurred through the rapid reduction of Fe(III) and the slow acidic dissolution, regardless of the pre-oxidation phase (Mäkinen et al., 2015; Hubau et al., 2020). The improvement of the pre-oxidation phase was not sufficient to accelerate the dissolution of metals since, as demonstrated by Hubau et al. (2020), the regeneration of Fe(III) by bio-oxidation of Fe(II) in batch mode is longer than the time required for the chemical dissolution of metals (acidic hydrolysis). By reducing the pre-oxidation phase to less than these 7 days, dissolution of metals could be accelerated. In our study, this was not reached, and the shortest duration of the pre-oxidation phase was not determined due to the difficulty of the operational mode: the study demonstrated that the time between the end of a batch and the next subculture should be reduced to a minimum to maintain short pre-oxidation phases. The 10-day pause between subcultures adversely affected microbial iron oxidation by extending the duration of the pre-oxidation phase. At the end of one subculture, iron is completely oxidized, resulting in a lack of electron supply for bacterial metabolism. It is therefore preferable to keep the periods of time between batch subcultures short, to maintain a better supply of electrons. Long periods before subculturing have a negative effect on the attempts to shorten the pre-oxidation phase. Nevertheless, this negative effect proved to be transient, as the cultures in this study eventually regained high oxidation performance after several subcultures.

A continuous-flow bioreactor offers an interesting alternative to avoid the pauses between batch subcultures by ensuring a constant supply of iron to be oxidized, from both the medium and the PCBs themselves. It also enables the cultures to be adapted to PCBs, as well as Fe(III) to be regenerated in parallel with its consumption, thus favoring the metal dissolution by Fe(III) over the acidic hydrolysis (Hubau et al., 2020). The challenge is to reach and maintain steady-state conditions since the start-up can be a limiting issue. Thanks to the successive batch subcultures before the start-up in continuous mode, Scenario III was the most suitable strategy. It maintained high and constant microbial oxidation of iron, indicating that Fe was maintained as Fe(III) despite the continuous addition of PCBs. It was also considerably faster than in a previous study where 67 days were required to reach high redox potentials in a steady state (Hubau et al., 2020). The start-up of the process until high microbial iron oxidation activity was now almost immediate. This is a great advantage for further development of the process and its scale-up.

## Conclusion

This study investigated the capacity of an FeOB culture to oxidize iron in PCB environments by means of indicators such as the redox potential and microbial growth. Exponential bacterial growth was observed and the bacteria oxidized 8 g L^–1^ of iron in the presence of a PCB leachate equivalent to 6% PCBs, which represented six times the metal content they were adapted to. However, the inhibiting effect of PCBs on microbial activity was evidenced by the delay in the bio-oxidation of Fe(II), i.e., a pre-oxidation phase. Successive subculturing shortened this delay by up to 2.5 times, reducing the inhibiting effect of PCBs in the bioleaching tests operated in batch mode. This strategy also enhanced the start-up of the continuous flow bioleaching reactor at 1% (w/v) PCBs. High microbial oxidation of iron was maintained by running several subcultures before switching to the continuous mode.

## Data Availability Statement

The raw data supporting the conclusions of this article will be made available by the authors, without undue reservation.

## Author Contributions

JA-G: conceptualization, methodology, investigation, writing—original draft, writing—review and editing, and visualization. AH: conceptualization, methodology, writing—review and editing, supervision, and project administration. CJ: conceptualization, methodology, writing—review and editing, and supervision. A-GG: conceptualization, resources, writing—review and editing, supervision, and funding acquisition. All authors contributed to the article and approved the submitted version.

## Conflict of Interest

The authors declare that the research was conducted in the absence of any commercial or financial relationships that could be construed as a potential conflict of interest.

## Publisher’s Note

All claims expressed in this article are solely those of the authors and do not necessarily represent those of their affiliated organizations, or those of the publisher, the editors and the reviewers. Any product that may be evaluated in this article, or claim that may be made by its manufacturer, is not guaranteed or endorsed by the publisher.
