# Supplementary material for: Bioleaching of E-Waste: Influence of Printed Circuit Boards on the Activity of Acidophilic Iron-Oxidizing Bacteria
Source: Front Microbiol. 2021 Aug 18;12:669738. doi: 10.3389/fmicb.2021.669738 (PMC8416503; doi:10.3389/fmicb.2021.669738)
Supplement: Supplementary file 1 [file Data_Sheet_1.docx]

Supplementary Material

# Supplementary Figures


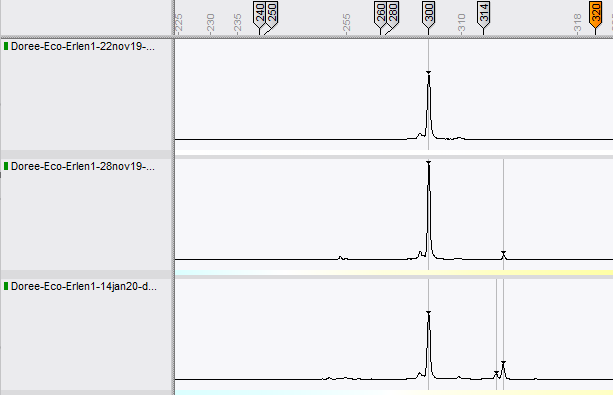

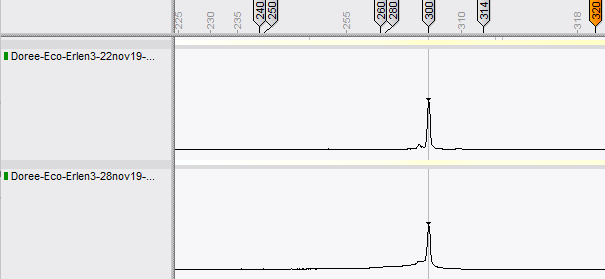

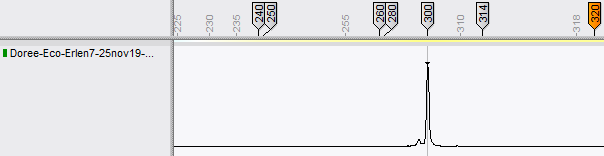

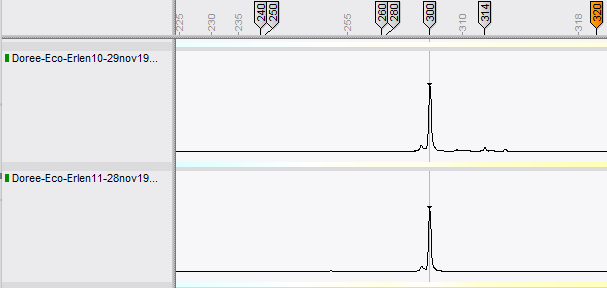

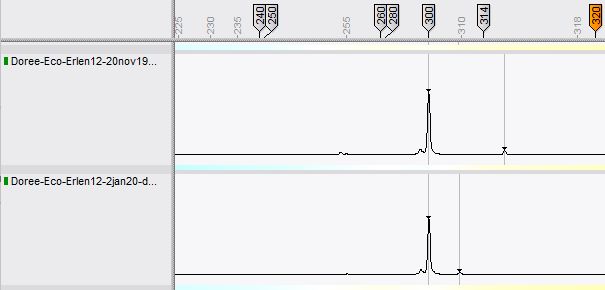


t_0_

2%

6%

4%

8%

**Supplementary Figure 1.** 16S rRNA gene fingerprints (CE-SSCP) of the microbial community in the inoculum and at the end of the tests in Erlenmeyer flasks at different leachate concentrations from 2% to 8% (w/v) PCBs. The gray reference lines correspond to the standard response of *Leptospirillum ferriphilum*.

**Supplementary Figure 2.** Follow up of the redox potential during the bioleaching at 1% (w/v) PCBs and 2% (w/v) PCBs in batch conditions with the adapted culture.

**Supplementary Figure 3.** Metal concentration and dissolution yield during bioleaching of 1% (w/v) and 2% (w/v) PCBs in bioleaching batch subcultures in 2.2 L-STRs. Experiments were performed 10% (v/v) of either the non-adapted or the adapted culture. The dissolution yields were calculated based on the estimated initial concentration of PCBs (Table 1) and the measured metal concentration in the leachate. Possible precipitation reactions were not taken into account.

**Supplementary Figure 4.** Bacterial concentration evolution during the subculturing at 2% (w/v) PCBs. The 1^st^ stage starts with the 3Cm medium while the 2^nd^ stage involves the addition of 2% (w/v) PCBs.

**Supplementary Figure 5.** Metal concentration and dissolution yield during bioleaching of 2% (w/v) PCBs in 2.2 L-STRs. Batch subcultures are represented as S0, S1, S2, S3, S4, and S5.


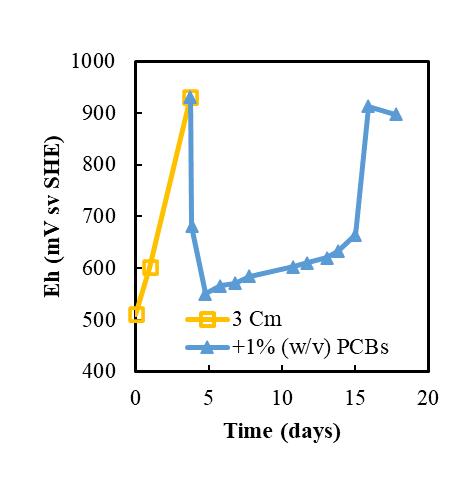


**A.**

**Supplementary Figure 6.** Redox potential of preconditioning batch systems before the continuous flow bioreactor systems. A) Scenario II with 1% (w/v) PCBs; B) Scenario III, where S0 represents the first batch at 2% (w/v) PCBs, S1 and S2 are the following subcultures with 50% (v/v) inoculum and 1% (w/v) PCBs.
